# A microRNA network functioning in the regulation of radiobiological effects

**DOI:** 10.1093/jrr/rrt157

**Published:** 2014-03

**Authors:** Xin Wu, Nan Ding, Wentao Hu, Jinpeng He, Lei Chang, Shuai Xu, Hailong Pei, Junrui Hua, Jufang Wang, Guangming Zhou

**Affiliations:** 1 Department of Space Radiobiology, Institute of Modern Physics, Chinese Academy of Sciences, 509 Nanchang Road, Lanzhou 730000, P.R. China; 2 University of Chinese Academy of Sciences, Beijing 100049, P.R. China

**Keywords:** microRNA, network, Dicer, BTG1, ionizing radiation

## Abstract

MicroRNA (miRNA), a small non-coding RNA molecule, is vital in genetic regulation, and miRNA pathway, which regulates gene expression through degradation or translational suppression of their target transcripts, is highly conservative in evolution.

Although profiles of miRNAs are different in various cell types and tissues, miRNAs have been considered as a crucial class of regulators in cellular response to ionizing radiation (IR). By carrying out a series of experiments, we have found that altered transcriptional regulation network composed of radiation-mediated miRNAs regulates the expression of their downstream target genes in most biological processes to control cell growth, cell cycle and apoptosis. For example, the newly identified miR-3928 negatively regulates the expression of Dicer, which has been validated by the luciferase assay and western blotting. Dicer is not only a key participant in responding to radiation, but also a critical factor for the maturation of miRNAs, suggesting that miR-3928 affects on the expression of other miRNAs through regulating Dicer. Among the miRNAs controlled by the Dicer, we reveal that miR-185 and miR-663 can efficiently suppress ATR and TGF-β1 expression, which are both important responders in the process of radiobiological effects. Further experiments reveal that the expression of Dicer is suppressed by miR-3928 induced by IR and consequently, the maturation of other miRNAs including miR-185 and miR-663 is inhibited, resulting in the abundantly enhanced expression of ATR and TGF-β1 respectively. This mechanism to hammer at fixing DNA damage or promote cells to apoptosis caused by IR has important implications in the decision of cell fates.

Moreover, it has been shown that the expression of BTG1 is characterized in response to factors that induce growth arrest and subsequent differentiation both *in vivo* and *in vitro*, affecting cellular physiological progresses of angiogenesis, follicular development and myoblast and B cell differentiation, through regulating cell growth, migration, cell cycle, apoptosis and differentiation. BTG1 gene is phylogenetically highly conserved in its coding and 3′-untranslated region (UTR), which is considered as a decisive element involved in regulation of BTG1 expression. We present evidence that BTG1 can be induced by IR and confirm that miR-454-3p, whose gene locates in the intron of Ska2 gene, can regulate BTG1 expression through directly binding to the 3′-UTR of BTG1 mRNA. These results point out that increased expression of BTG1 caused by the down-regulation of miR-454-3p in case that IR modulates endogenous activity of PRMT1, a BTG1-binding partner, which can methylate endogenous transcription factors to change gene expression pattern and reply radiostilumation. An inverse relationship between the levels of expression of BTG1 and miR-454-3p reveals that there exists a new pathway in response to IR stimulation. Furthermore, cell growth will be transiently increased by the knockdown of BTG1 by transfecting BTG1 siRNA or miR-454-3p mimic. However, the apoptotic state of cells can be tested after 2 days. Down-regulation of BTG1 by miR-454-3p increases the sensitivity of human renal cell carcinoma 786-O cells to IR-induced apoptosis, suggesting that BTG1 could serve as a terget for sensitizing renal carcinoma to standard radiotherapy.

Taken together, all these data indicate that alteration of miRNA expression is evident in the cellular response to IR. MiR-3928, miR-185, miR-663 and miR-454-3p may constitute a complex network contributing to the regulation of radiobiological effects. It is apparent that the study of radiation-related miRNAs is beneficial to qualitatively and quantitatively modulating radiobiological effects, and also in favor of the advanced research of miRNA functions.

